# Exploring the Molecular Mechanisms of Resistance to Prochloraz by *Lasiodiplodia theobromae* Isolated from Mango

**DOI:** 10.3390/jof10110757

**Published:** 2024-10-31

**Authors:** Rui He, Jinlin Liu, Pengsheng Li, Yu Zhang, Xiaoyu Liang, Ye Yang

**Affiliations:** 1 Sanya Institute of Breeding and Multiplication, Hainan University, Sanya 572025, China; harry8098@163.com (R.H.); ljl777xxx@163.com (J.L.); yangyewuxi@163.com (P.L.); yuzhang_rain@hainanu.edu.cn (Y.Z.); 2 School of Life and Health Sciences, Hainan University, Haikou 570228, China; 3 School of Tropical Agriculture and Forestry, Hainan University, Haikou 570228, China

**Keywords:** *Lasiodiplodia theobromae*, target resistance, metabolic resistance, cytochrome P450, overexpression, molecular docking

## Abstract

Mango stem-end rot caused by *Lasiodiplodia theobromae* is a major postharvest disease in China. Prochloraz is commonly used for disease control in mango orchards and in storage. However, prochloraz resistance has been detected in *L. theobromae*. This study aimed to explore the underlying mechanisms responsible for prochloraz resistance in *L. theobromae*. The results show that no point mutation in the target gene *LtCYP51* of the prochloraz-resistant *L. theobromae* strain was detected, but the expression was upregulated significantly. Additionally, the full-length sequences of the cytochrome P450 gene *CYP55A3* were successfully amplified and identified from *L. theobromae*, and the qRT-PCR results confirm that *CYP55A3* was significantly upregulated after treatment with prochloraz. The knockout mutant of the *CYP55A3* presented significantly lower gene expression levels than the wild-type strain HL02, with a 16.67-fold reduction, but a 1.34-fold reduction in P450 activities and a 1.72-fold increase in the accumulation of prochloraz in the mycelia. Treatment with the P450 enzyme inhibitor significantly synergized with the prochloraz toxicity. The wild-type strain was highly resistant to pyraclostrobin and carbendazim; similarly, the sensitivity of the knockout mutant to pyraclostrobin and carbendazim also notably increased. There was no significant difference between the wild-type strain and the gene-complemented strain. The homology model and molecular docking analysis provide evidence that prochloraz interacts with the protein structure of CYP55A3. These findings suggest that the overexpression of the target gene *LtCYP51* and the detoxification gene *CYP55A3* were involved in the molecular mechanisms of resistance to prochloraz by *L. theobromae*.

## 1. Introduction

Mango (*Mangifera indica* L.) is a globally renowned tropical fruit that is widely distributed in tropical and subtropical regions. It is rich in vitamins, antioxidants, and minerals and has important economic value [1]. China ranks second in the world in mango plantation area size, with Hainan province being one of the main mango-producing regions in China, exceeding 68,667 hectares in 2024. Mango, the largest crop cultivated in Hainan, ranks second in the gross value of agricultural production. Stem-end rot (SER) disease caused by *Lasiodiplodia theobromae* is one of the major postharvest diseases that results in considerable loss during storage and transportation, and the incidence of SER is about 30% [2]. This pathogen has latent infection characteristics; it can infect fruits during the young fruit period and remains latent [2,3]. Disease spots emerge and rapidly expand when the fruit ripens, causing fruit rot and leading to severe economic losses. *L. theobromae* is the causal agent of numerous plant diseases in a wide variety of hosts, with a worldwide distribution. It causes rapid decay of the fruits of avocado, banana, orange, papaya, and longan [4,5,6]. Besides SER, it is also associated with branch dieback, gummosis, and root rot [7,8,9]. With the widespread use of fungicides for disease control in mango, some pathogens are becoming increasingly resistant to fungicides in Hainan. The resistance of *L. theobromae* from mango to carbendazim has increased dramatically over the past decade [10]. The resistance of this pathogen to difenoconazole and prochloraz has also been reported in recent years [11,12].

Demethylation inhibitor (DMI) difenoconazole, myclobutanil, prochloraz, etc., are widely used to control preharvest and postharvest diseases in mango in China [13]. The DMI fungicide, which prevents the biosynthesis of ergosterol by inhibiting 14-α-demethylase, can effectively inhibit the growth of mycelia. The 14α-demethylase (*CYP51*) gene has been identified as a good target for fungicides [14]. Recently, more DMI-resistant strains have been identified and studied. The resistance mechanisms can be divided into target-site resistance (TSR) and nontarget-site resistance (NTSR) [15,16]. For example, Chen et al. (2024) reported that *Botrytis cinerea* was examined for its resistance to pyrisoxazole, and mutations in the *CYP51* gene and the induced overexpression of efflux proteins appeared to be involved in conferring resistance [17]. TSR involves the target genes; many studies support the idea that DMI fungicides prompt the development of resistance in pathogens through the mutation and/or upregulation of the *CYP51* gene [18,19]. NTSR involves detoxification enzymes and transporter proteins, also known as metabolic resistance [9]. Metabolic resistance represents a crucial mechanism of drug resistance, yet the vast majority of studies have focused on insecticide and herbicide resistance. The cytochrome P450 monooxygenase (P450) superfamily is a diverse enzyme family and is a common and important detoxification enzyme in all living organisms. Studies on the resistance of pests and weeds to pesticides have confirmed that P450s are related to resistance; Wang et al.’s (2018) study revealed that the transcription levels of five P450 genes in the larvae of insecticide-resistant *Spodoptera exigua* markedly increased after treatment with β-cypermethrin [20]. The P450-mediated pyrethroid resistance in *Aedes aegypti* resulted in cross-resistance to both methyl paraoxon and fenitrothion [21]. The mechanism of herbicide resistance is the overexpression of *CYP81A68* in *Echinochloa crus-galli* [22]. In comparison with pests and weeds, there are only a few reports of pathogens degrading exogenous compounds via the overexpression of detoxification proteins. Sang et al. (2018) reported that P450s play crucial roles in the xenobiotic detoxification system of *Sclerotinia homoeocarpa* [23]. Zhang et al. (2020) reported that *Magnaporthe oryzae* upregulated the expression of three P450 genes to increase resistance to novel pyrimidine amines [24]. The overexpression of the P450 gene contributed to resistance in *Rhizoctonia solani* via a detoxification pathway [25].

The metabolic resistance of fungicides has gradually become a concern in recent years and is a very innovative research field. DMI-resistant strains of *L. theobromae* have been found in mango in China. Previous studies revealed no mutations in the target gene *CYP51* in strains resistant to DMI fungicides [11,12]. In addition, RNA-seq analysis indicated that the differentially expressed genes in *L. theobromae* strains were primarily enriched in metabolism-related pathways, and the expression levels of some P450 genes in the resistant strain were upregulated after treatment with prochloraz [26]. It is still uncertain whether the prochloraz resistance was associated with P450-mediated detoxification. Therefore, this study attempted to explore the prochloraz resistance mechanism of *L. theobromae* from both sides of the target gene and the detoxification gene. The approach adopted involved (1) determining the DNA sequence and expression of the target gene *LtCYP51* in the resistant strain; (2) identifying and measuring the expression of a P450 gene *CYP55A3* in *L. theobromae*; (3) carrying out the gene knockout of the *CYP55A3* gene via homologous recombination and verifying the function of this gene related to prochloraz resistance; (4) determining the synergism of detoxifying enzyme inhibitors mixed with prochloraz and testing fungicide sensitivity; and (5) explaining the binding affinity of prochloraz with CYP55A3 through molecular docking simulations. These results offer a broader perspective for investigating the molecular mechanisms of prochloraz resistance and provide a theoretical basis for the prevention and control of *L. theobromae* and the use of fungicides.

## 2. Materials and Methods

### 2.1. Test Strains and Chemicals

The prochloraz-sensitive strain DF04 and the prochloraz-resistant strain HL02 were obtained from diseased fruit with typical SER symptoms in Hainan province, China, and were previously isolated, identified and preserved in our laboratory (School of Tropical Agriculture and Forestry, Hainan University in 2019). The EC_50_ value of Hl02 was 8-fold higher than that in the DF04 [26]. Pure strains were grown on PDA media, with subsequent incubation at 28 °C for 3 days.

The following five technical fungicides were used: prochloraz, carbendazim, pyraclostrobin, fludioxonil and iprodione. To prepare stock solutions, prochloraz (97% a. i.), fludioxonil (98% a. i.) and iprodione (97% a. i.) were dissolved in 100% acetone to obtain 5 × 10^3^ μg/mL solutions. Carbendazim (97.2% a. i.) was dissolved in 0.1 M hydrochloric acid to obtain a 5 × 10^4^ μg/mL solution. Pyraclostrobin (97% a. i.) was dissolved in 100% acetone to obtain a 1 × 10^4^ μg/mL solution. The cytochrome P450-dependent monooxygenase-inhibitor 1-aminobenzotriazole (1-ABT) was dissolved in dimethyl sulfoxide (DMSO) and added to 0.1% Tween-80 water to prepare a 2000 µg/mL solution. All the solutions were stored at 4 °C in the dark.

### 2.2. Cloning, Sequencing and Quantitative Expression of LtCYP51 Gene

The tested strains were grown on PDA at 28 °C for 3 days. Then, the strains were inoculated into 100 mL of PDB and incubated at 150 rpm and 28 °C for 24 h. The mycelia of each strain were removed, and the total genomic DNA was extracted via the E.Z.N.A.^®^ HP Plant DNA Mini Kit (Omega Bio-Tek, Norcross, GA, USA). Primers LtCYP51-F1/LtCYP51-R1 and Per-1F/Per-1F (Table S1) were used to amplify the *LtCYP51* coding sequence and promoter. The cloned fragments were connected to the *pESI-blunt* vector (YEASEN Biotech Co. Ltd., Shanghai, China) plasmid, transformed into *DH5α* cells, and sequenced at NanShan Science and Technology Co., Ltd. (Haikou, China) using the vector primers M13F and M13R (Table S1).

After culturing *L. theobromae* in PDB for 24 h, prochloraz was added until the final concentration reached 10 µg/mL, and the mixture was incubated for 12 h. The mycelia of each strain were collected, and the total RNA sample was preliminarily judged for contamination and degradation by 1% agarose gel electrophoresis. After the RNA samples passed the quality inspection, they were enriched for mRNA using Oligo (dT) magnetic beads, fragmented, and used as a template to synthesize cDNA. The specific primers were designed via Oligo 7 (Molecular Biology Insights Inc., Colorado Springs, CO, USA). Primers RT-CYP51-F/R were used to quantify RT-PCR (qRT-PCR) to analyze *LtCYP51* expression (Table S1). A total volume of 20 µL was added with 2 × ChamQ Universal SYBR qPCR Master Mix (Vazyme Biotech Co., Ltd., Nanjing, China) to amplify the genes in the qTOWER3 G REAL-TIME PCR thermocycler (Analytik Jena AG, Jena, Germany). The reference gene *β-actin* was used as the internal control for RNA quality. The relative expression levels of genes were determined utilizing the 2^−∆∆Ct^ method. Each treatment has three biological replicates and three technological replicates.

### 2.3. Identification and Quantitative Analysis of L. theobromae CYP55A3 Gene

According to previous results about transcriptome analysis of prochloraz-resistant strains of *L. theobromae* [20], the significantly upregulated P450 gene *CYP55A3* was selected for further functional characterization in this study, which is highly related to nitrogen metabolism based on KEGG pathway (ko00910). The primers CYP55A3-F/CYP55A3-R and CYP55A3-CDS-F/CYP55A3-CDS-R (Table S1) were designed to amplify the *CYP555A3* coding sequence. The PCR mixture contained 25 μL of 2 × Phanta Max Master Mix, 1 μL of template DNA, 2 µL of each primer (10 µM), and ddH_2_O such that the final reaction volume was 50 μL. A gel recovery kit (Omega Bio-Tek, Norcross, GA, USA) was used to recover the bands. The amplified fragments were sequenced by Sangon Biotech Co., Ltd. (Shanghai, China). Nucleotide sequences were edited with BioEdit software (v7.2.5) and analyzed with BLAST in the GenBank database. The protein sequences were predicted by BioXM 2.7.1. The phylogenetic tree was constructed using neighbor-joining in MEGA-X (bootstrap values on the basis of 1000 replicates), two sequences obtained from our study and six reference CYP55A3 protein sequences obtained from GenBank (Table S2). The physico-chemical properties were predicted using the ExPASy-ProtParam tool. The transmembrane domain was predicted by TMHMM Serverv 2.0.

Primers RT- CYP55A3-F/R were used for qRT-PCR to analyze *CYP55A3* expression level (Table S1). The methods are described in Section 2.2.

### 2.4. Construction of Vector and Genetic Transformation

Primer Premier 5.0 was used to design amplification primers for the upstream and downstream fragments of *CYP55A3* (Table S1). The hygromycin phosphotransferase gene was used as the resistance screening gene for the *CYP55A3* gene knockout vector. The upstream and downstream fragments of *CYP55A3* were amplified using HL02 DNA as a template. A gel recovery kit (Omega Bio-Tek, Norcross, GA, USA) was used to recover the bands. The vector *pUC19* was digested with Bam HI and Hind III restriction enzymes and subsequently recovered. The ClonExpress Ultra One Step Cloning Kit (Vazyme Biotech Co., Ltd., Nanjing, China) was used to connect the upstream and downstream fragments of the *CYP55A3* gene with the enzyme-digested vector skeleton via overlap PCR. For the complementation experiment of the *CYP55A3*, the *CYP55A3* gene fragment was amplified using CDS-F/R, and the pBARGPE1-G418 vector and the *CYP55A3* gene fragment were digested with Bam HI and Eco RI restriction enzymes and recovered. The resulting products were then transformed into *Escherichia coli* and the recombinant plasmid was extracted. The recombinant plasmids were transformed into *L. theobromae* by PEG-mediated protoplast genetic transformation to obtain the gene knockout transformant ∆*Ltcyp55a3* and the gene complementation transformant ∆*Ltcyp55a3*/*LtCYP55A3*. After screening the transformants, the transformants were inoculated onto PDA containing antibiotics (corresponding to the resistance marker) and subcultured 2‒3 times to obtain stable genetic transformants.

### 2.5. Detection of the Gene Expression Levels, P450 Activity and Prochloraz Accumulation in the Mycelia

Primers RT-CYP55A3-F/R were used for qRT-PCR to detect the gene expression levels of the *CYP55A3* gene for the wild-type strain HL02, the ∆*Ltcyp55a3* deletion mutant and the ∆*Ltcyp55a3/LtCYP55A3* strain (Table S1). The methods are described in Section 2.2.

All the tested strains were cultured in PDB media at 28 °C and 145 rpm for 24 h. Then, the cultures were treated with 10 µg/mL prochloraz for 12 h, and the mycelia were collected and stored at −80 °C. All the samples were homogenized with phosphate-buffered saline (0.04 M, pH 7.4). The supernatant was collected as the enzyme extract. A microbial cytochrome P450 (CYP450) ELISA Kit (Hengyuan Biotechnology Co., Ltd., Shanghai, China) was used to determine P450 activity, using 7-ethoxycoumarin as a substrate, which is converted to 7-hydroxycoumarin (7-HC) by P450. The absorbance value of 7-HC in each sample is determined at 450 nm, and then P450 activity was calculated from a standard curve of 7-HC. For each strain, three biological replicates and two independent replicates were conducted.

All the tested strains were cultured in PDB at 28 °C and 145 rpm for 24 h. Then, the cultures were treated with 10 µg/mL prochloraz for 6 h, and the mycelia were collected and stored at −80 °C. The samples were extracted with acetonitrile and analyzed via ultrahigh-performance liquid chromatography‒tandem quadrupole mass spectrometry (UPLC-MS/MS). The column used was a ZORBAX Eclipse Plus C18 (50 × 3.0 mm, 1.8 µm) at 40 °C, with a flow rate of 300 mL/min. The mobile phase consisted of acetonitrile and 0.1% formic acid in water (gradient elution). A standard curve was prepared using prochloraz at concentrations ranging from 1 to 100 ng/mL. Recovery tests were conducted with 10–40 mg/kg concentrations of prochloraz.

### 2.6. Prochloraz Toxicity and Synergism by P450 Enzyme Inhibitors

The effects of a P450 detoxifying enzyme inhibitor (1-ABT) on the toxicity of prochloraz to resistant strains and transgenic strains were determined via the mycelial growth rate method. Activated resistant strains of *L. theobromae* were inoculated in PDA media supplemented with prochloraz, 1-ABT, and prochloraz-1-ABT mixture (10 µg/mL each). The cultures were incubated at 28 °C for 30 h, and the diameter of each colony was measured to calculate the percentage of mycelial growth inhibition. Each treatment was performed in triplicate, and the experiment was repeated three times independently. The synergistic ratio (SR) was calculated by dividing the inhibition rate in the presence of the synergist by that of prochloraz alone. An SR value >1 indicates a synergistic effect of mixtures [27].

### 2.7. Sensitivity to Five Fungicides

Bioassays to assess the sensitivity of the ∆*Ltcyp55a3* deletion mutant and ∆*Ltcyp55a3*/*LtCYP55A3* strains to five fungicides (prochloraz, carbendazim, pyraclostrobin, fludioxonil and iprodione) were carried out via the mycelial growth rate method. First, PDA media plates with different concentrations of each fungicide were prepared. The final solvent concentration in the medium was 1%, and control media with equal amounts of solvent but without fungicide were used. The mycelial discs (4 mm in diameter) of the strains were subsequently transferred to PDA plates containing fungicide. The tested strains were incubated at 28 °C for 30 h, and the diameter of each colony was measured to calculate the percentage of mycelial growth inhibition. Each treatment was repeated three times, and two independent replicates were conducted. The inhibition rates were converted to probability values and concentrations were log 10-transformed before a line regression model was used. The EC_50_ values were calculated by the regression equation.

### 2.8. Homology Modeling of CYP55A3 and Molecular Docking of Prochloraz

Molecular docking was performed to determine whether CYP55A3 plays a significant role in prochloraz metabolism. The 3D structure of prochloraz was acquired from the PubChem database (https://pubchem.ncbi.nlm.nih.gov/ (26 June 2024). First, Swiss modeling (https://swissmodel.expasy.org/ (27 June 2024) was used for homology modeling to obtain the 3D structure of the protein. The 3D protein model was subsequently evaluated by the Ramachandran plot. The AutoDock Vina program was then used to conduct molecular docking simulations to elucidate the interaction mechanisms between prochloraz and CYP55A3. Molecular docking analysis was performed using the Lamarckian genetic algorithm and 100 independent runs per ligand to evaluate binding energy of ligands with receptor proteins. After this analysis, the protein–ligand docked complexes with the lowest binding energy were generated and utilized to obtain 3D visualization via PyMOL tools. The details of the interaction between prochloraz and P450 (CYP55A3) were provided by LigPlot software version 2.2 (https://www.ebi.ac.uk/thorntonrv/software/LigPlus/download2.html (10 July 2024).

### 2.9. Statistical Analysis

Each treatment was performed in triplicate, and the experiment was repeated two or three times independently. All data were subjected to ANOVA and Duncan’s multiple range test using the statistical software SPSS Statistics 24.0.

## 3. Results

### 3.1. Sequencing and Expression Level of LtCYP51 Gene

The nucleotide sequences of the *LtCYP51* gene exhibited 99% identity with that of *L. theobromae* AM2As (GenBank accession number MK107983.1). This gene fragment encodes 523 amino acids and contains two introns, each 49 base pairs long, located at nucleotide positions 247 and 494, respectively. Two strains were analyzed for the sequence of *LtCYP51* gene and their upstream regions. Compared with sensitive strain DF04, no mutation was found in the resistant strains HL02. The upstream regions exhibited complete identity among all tested strains, and no mutations or insertions were identified in the promoter region of the *LtCYP51* gene.

**Figure 1:**
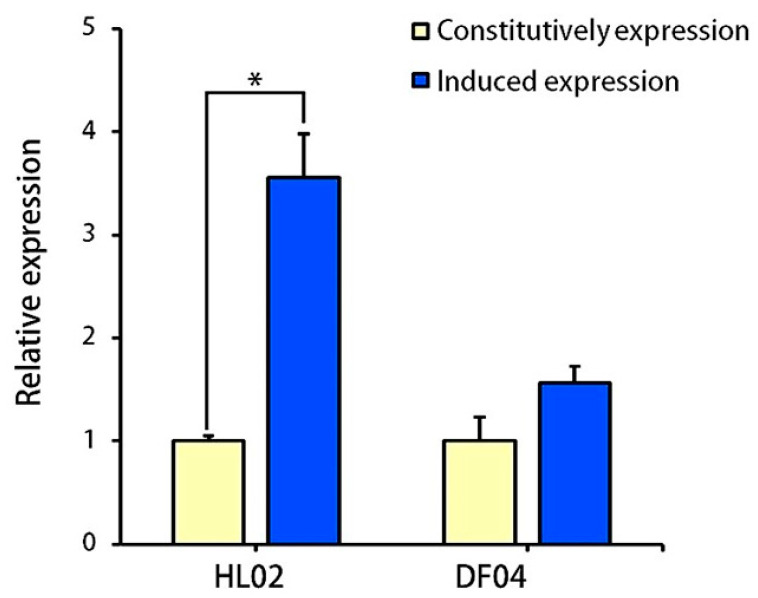
Constitutive and fungicide-induced expression of the target gene *LtCYP51* in the *L. theobromae* strains (HL02 and DF04) before and after treatment with prochloraz. * above the bars indicates significant differences (*p* ≤ 0.05).

The expression level of *LtCYP51* gene in the resistant strain increased significantly after prochloraz treatment (Figure 1). In the prochloraz-resistant strains HL02, the relative expression level of *LtCYP51* was significantly greater than in the sensitive strain DF04 (3.36-fold) (*p* ≤ 0.05), suggesting that the resistance of *L. theobromae* to prochloraz might be related to the fungicide-induced overexpression of the *LtCYP51* gene.

### 3.2. Identification and Expression of L. theobromae CYP55A3 Gene

**Figure 2:**
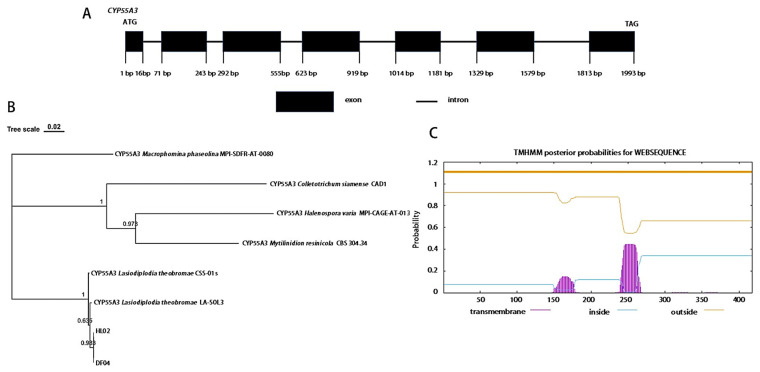
Sequence analysis of resistant *L. theobromae CYP55A3* gene and protein. (**A**) DNA sequence and structural characteristic; (**B**) nearest-neighbor analyses of CYP55A3; (**C**) transmembrane domain structure.

The full-length sequences of *CYP55A3* gene were successfully amplified from DF04 and HL02 strains, 2000 bp or so in length and containing 7 exons (Figure 2A). The phylogenetic tree was constructed via the NJ method, and the sequence analysis reveal that the CYP55A3 protein from DF04 and HL02 obtained in this study clustered into the clade of CYP55A3 from *L. theobromae* (Figure 2B). The CYP55A3 protein sequence was 99.5% to 100% homologous to *L. theobromae* CYP55A3 (KAB2574247.1 and KAF9635644.1). The results of bioinformatics analysis show that the molecular weight was 49.40 kDa, the theoretical isoelectric point was 5.03, the instability index was 38.24, the CYP55A3 protein had transmembrane domain structure (Figure 2C) and no signal peptide sequence and was not a secretory protein.

**Figure 3:**
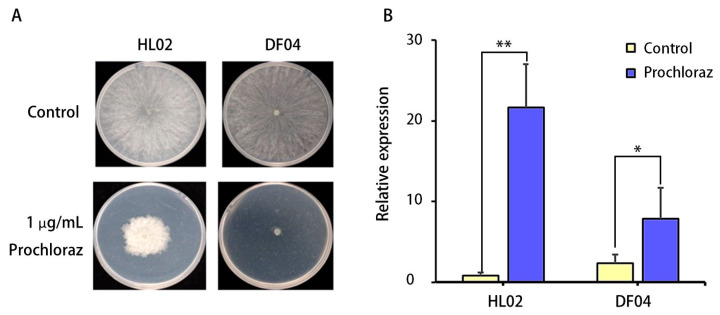
Difference of gene expression of the P450 gene *CYP55A3* in the *L. theobromae* before and after treatment with prochloraz. (**A**) The mycelial growth of prochloraz-resistant and prochloraz-sensitive strains; (**B**) *CYP55A3* gene expression of resistant strain HL02 and sensitive strain DF04. * above the bars indicates significant differences (*p* ≤ 0.05); ** above the bars indicates significant differences (*p* ≤ 0.01).

The sensitive strain DF04 did not grow in PDA media with 1 µg/mL prochloraz (Figure 3A). The qRT‒PCR results show that *CYP55A3* genes of the two strains were significantly upregulated after treatment with prochloraz (Figure 3B) (*p* ≤ 0.05). In the prochloraz-resistant strain HL02, the relative expression level of the *CYP55A3* was significantly greater than in the sensitive strain DF04.

### 3.3. Vector Construction and Usage for Gene Knockout

**Figure 4:**
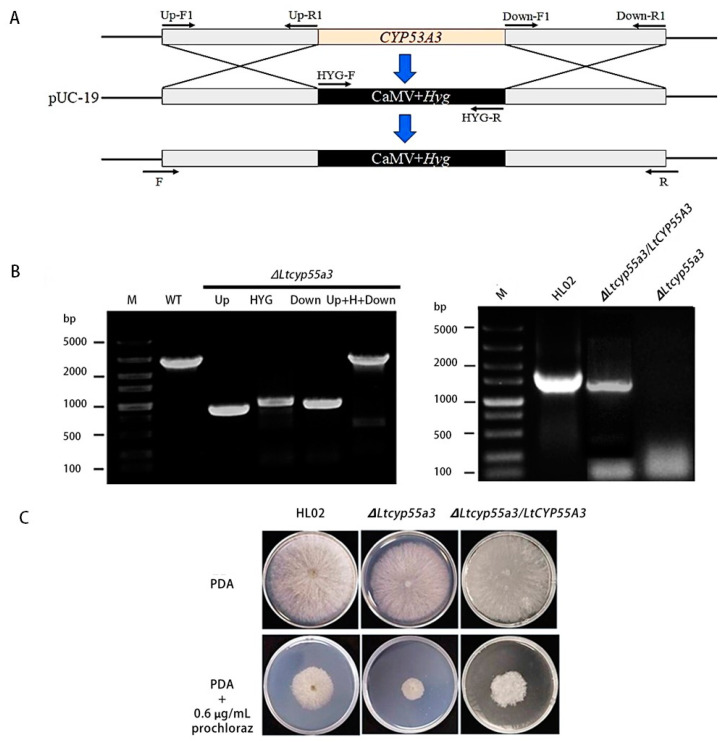
Generation of the *CYP55A3* gene deletion mutants in *L. theobromae*. (**A**) Strategy for the construction of gene replacement fragment; (**B**) verification of *CYP55A3* gene knockout mutants by PCR amplified with primers UP (UpF1/UpR1), primers Down (DownF1/DownR1), primers HYG (HYG-F/HYG-R), primers UP+H + Down (F/R); (**C**) the mycelial growth of the wild-type and mutant strains after treatment with prochloraz. M: marker, WT: HL02 strain, ∆*Ltcyp55a3*: gene deletion mutants, ∆*Ltcyp55a3*/*LtCYP55A3*: gene-complemented mutants.

To confirm the involvement of *CYP55A3* in metabolic resistance to prochloraz in *L. theobromae*, a transgenic HL02 strain with knockout and complementation of *CYP55A3* were constructed by homologous recombination knockout technology; the strategy for the construction of the gene replacement fragment is shown Figure 4A. The upstream and downstream parts were fused by Overlap PCR. The gene-knockout and gene-complemented mutants were confirmed by PCR and DNA sequencing (Figure 4B). The sensitivity of the knockout strain to prochloraz decreased significantly, but that of the complemented strain was similar to that of the wild-type strain (Figure 4C). These results indicate that positive transformants of the knockout (∆*Ltcyp55a3*) and complemented (∆*Ltcyp55a3*/*LtCYP55A3*) strains were successfully generated from the resistant strain HL02.

### 3.4. Gene Expression Level, P450 Activity of Transformants and Prochloraz Accumulation Amount in the Mycelia

**Figure 5:**
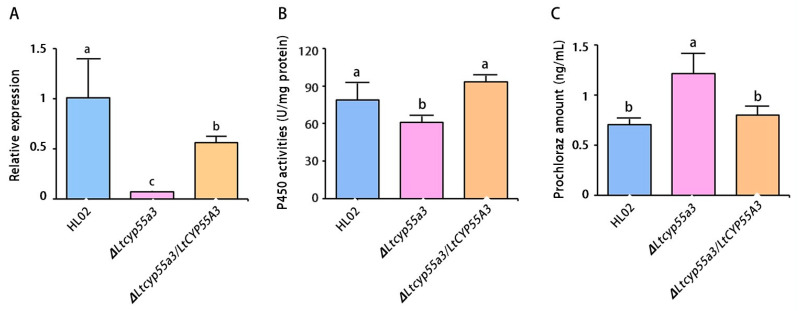
Comparison of the wild-type and mutant strains after treatment with prochloraz. (**A**) The expression of *CYP55A3* gene of ∆*Ltcyp55a3* mutant was significantly decreased; (**B**) the P450 activities of ∆*Ltcyp55a3* mutant were significantly decreased; (**C**) the accumulation of prochloraz of ∆*Ltcyp55a3* mutant was significantly increased. Different letters above the bars indicate significant differences (*p* ≤ 0.05).

The gene expression level of the knockout mutant ∆*Ltcyp55a3* was significantly lower than that of the wild-type HL02 strain (16.67-fold) and the gene-complemented strain Δ*Ltcyp55a3*/*LtCYP55A3* (9.16-fold) after treatment with prochloraz (Figure 5A). Accordingly, the P450 activities of both the HL02 and Δ*Ltcyp55a3*/*LtCYP55A3* strains were significantly greater than that in the sensitive ∆*Ltcyp55a3* strain after 12 h of incubation (*p* ≤ 0.05) (Figure 5B). Compared with that of the *∆Ltcyp55a3* strain, the P450 activities were increased by 1.31- and 1.57-fold in strains HL02 and *∆Ltcyp55a3/LtCYP55A3*, respectively.

In the range of 1.00 to 100.00 ng/mL, UPLC-MS/MS analysis demonstrated a good linear relationship between the instrument response value and the mass concentration, with a correlation coefficient greater than 0.999. The percent recovery of prochloraz ranged from 80.9% to 84.8% when the concentration was increased from 10 to 40 mg/kg. The accumulation of prochloraz in the mycelia treated with prochloraz was measured via UPLC‒MS/MS analysis. The results implied that the accumulation of prochloraz in the ∆*Ltcyp55a3* strain was significantly greater than that in both the HL02 and the Δ*Ltcyp55a3*/*LtCYP55A3* strains after prochloraz treatment for 6 h (*p* ≤ 0.05) (Figure 5C). Compared with that in the ∆*Ltcyp55a3* strain, the accumulation of prochloraz was reduced by 1.72- and 1.51-fold in strains HL02 and ∆*Ltcyp55a3*/*LtCYP55A3*, respectively.

### 3.5. Synergism of Enzyme Inhibitors to Prochloraz

The P450 enzyme inhibitor 1-ATB and prochloraz were investigated by co-application to determine the role of P450s in metabolic resistance. The synergistic ratios (SRs) of the HL02, ∆*Ltcyp55* and ∆*Ltcyp55a3*/*LtCYP55A3* strains were 1.23, 0.88 and 1.24, respectively (Table 1). The inhibition rate increased significantly when treated with the combination of prochloraz and 1-ATB for strains with resistance to fungicide. The results suggest that treatment with 1-ATB significantly promoted prochloraz toxicity, and P450 may play a role in the development of prochloraz resistance.

### 3.6. Sensitivity of the Five Fungicides of Transformants

**Figure 6:**
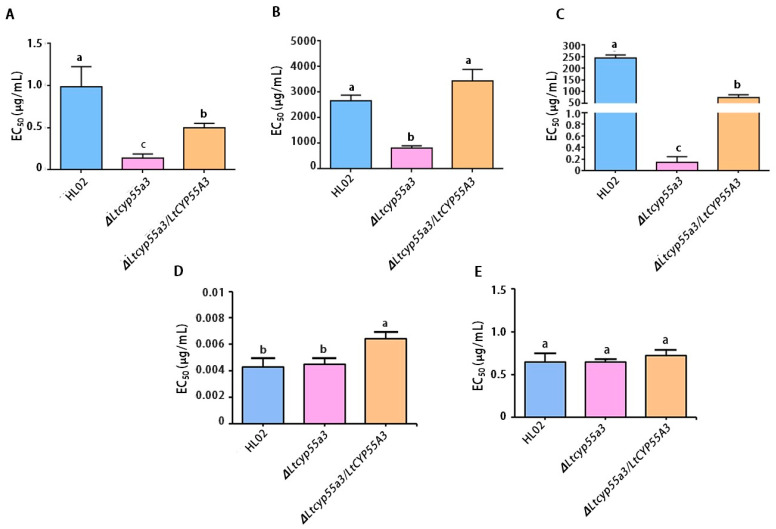
Change in the EC_50_ values of the wild-type and mutant strains for five fungicides. (**A**–**C**) The knockout mutant ∆*Ltcyp55a3* became more susceptible to prochloraz, carbendazim, pyraclostrobin; (**D**,**E**) there was not significant difference between the wild-type and mutant strains to fludioxonil and iprodione. Different letters above the bars indicate significant differences (*p* ≤ 0.05).

The results of the sensitivity test reveal that the knockout mutant ∆*Ltcyp55a3* was significantly more sensitive to prochloraz than the wild-type strain HL02 and the complemented ∆*Ltcyp55a3*/*LtCYP55A3*. Among them, the ∆*Ltcyp55a3* strain had an EC_50_ value for prochloraz that was 9.17-fold lower than that of HL02, and the ∆*Ltcyp55a3*/*LtCYP55A3* strain had an EC_50_ value for prochloraz that was 4.45-fold greater than that of ∆*Ltcyp55a3* (Figure 6A).

In addition, four fungicides can be divided into two groups based on their EC_50_ values. One group included the HL02 strain, which was highly resistant to carbendazim and pyraclostrobin. Thus, the EC_50_ values of the *∆Ltcyp55a3* strain were 1.78- to 3387.43-fold and 5.36- to 1100.57-fold lower than those of the HL02 and ∆*Ltcyp55a3*/*LtCYP55A3* strains, respectively. The sensitivity of the knockout mutant strain to pyraclostrobin and carbendazim obviously increased, and resistance markedly decreased. This variation in the EC_50_ values was similar to that of prochloraz (Figure 6B,C). And the other group including HL02 strain was sensitive to fludioxonil and iprodione, and the EC_50_ values of ∆*Ltcyp55a3* were not significantly different from those of HL02 (Figure 6D,E). These results demonstrate that the *CYP55A3* gene is involved in the cytochrome P450-mediated metabolism of prochloraz, pyraclostrobin and carbendazim, leading to the resistance of *L. theobromae* to prochloraz and the other two fungicides.

### 3.7. Molecular Docking Analysis of Prochloraz with CYP55A3

**Figure 7:**
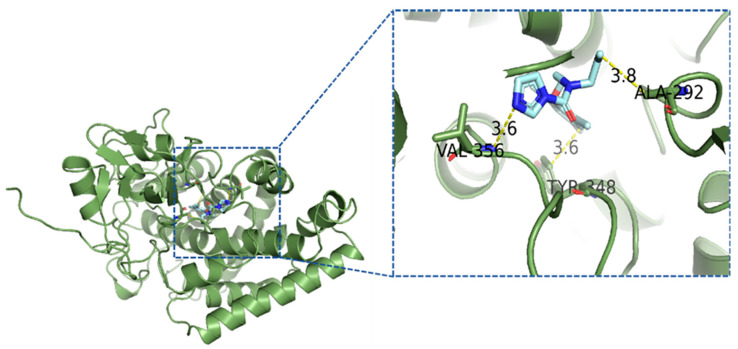
Binding modes and molecular interactions of prochloraz to P450 protein CYP55A3. Prochloraz is in cyan color, parts of protein in green color; on the right, hydrophobic interactions, hydrogen bond and interacting amino acid residues are shown; yellow dotted line denotes interatomic distances.

The 3D structure of CYP55A3 was derived by homology modeling. The 3D protein model was evaluated by the Ramachandran plot, and the results reveal that 94.7% of the residues were in the most favored regions, 4.7% of the residues were in additional allowed regions, and 0.5% of the residues were in generously allowed regions (Figure S1). The binding modes of prochloraz were determined by molecular docking simulations. The docking results show that prochloraz had good affinity for CYP55A3, with binding energies of −6.3 kcal/mol, and both the RMSE (<0.06) and relative root mean square error (RRMSE < 1.895) were low, indicating that the simulation results of the model were reliable. The details of the interaction between prochloraz and 3D CYP55A3 provided by Ligplot software are given in Figure 7. Prochloraz interacted with three amino acid residues located in the P450 enzyme CYP55A3 (labeled in green) in the model. In the docked complexes, prochloraz had hydrophobic interactions with TYR348 and ALA29 and the lengths of the coordination bonds between prochloraz and the two residues were 3.6 and 3.8 Å, respectively. Additionally, prochloraz formed hydrogen bond interactions with VAL356, whose hydrogen bond distance was 3.6 Å (Figure 7). The molecular docking results indicated that prochloraz had good binding activity with CYP55A3.

## 4. Discussion

More fungicide-resistant *L. theobromae* strains have been detected and studied in mango and papaya in recent years [6,11,12,13]. DMI fungicide resistance is often related to target gene mutations and/or overexpression of efflux proteins [19,28,29]. Wang et al. (2023) reported that the upregulated expression of *ABCG* gene was involved in prochloraz resistance of *L. theobromae* [13]. Our studies also exhibited that no point mutation existed in the *LtCYP51* gene of prochloraz-resistant *L. theobromae* strain, but the expression levels of the *LtCYP51* gene were significantly greater than that in the sensitive strain. As a target protein of prochloraz, the CYP51 enhancement can also enhance fungicide resistance. Are there other mechanisms of resistance for *L. theobromae*?

Recent studies have focused on the molecular mechanism of fungicide resistance in NTSR. Among them, P450s mediating the metabolic resistance pathway, most likely through enhanced detoxification of xenobiotics, have been confirmed in previous disease, pest and weed studies [24,30,31]. In our previous studies, the upregulated expression of some P450 genes in resistant *L. theobromae* strains was found by transcriptomic analysis [26]. Based on this, the P450 gene *CYP55A3*, which is highly related to nitrogen metabolism, was selected for further functional characterization. Here, P450 gene *CYP55A3* was cloned and identified from *L. theobromae.* It is a member of the CYP55 subfamily, which catalyzes the co-denitrification reaction and additionally exhibits NADH-peroxidase activity [32]. At first, qRT-PCR analysis results showed that the relative expression level of the prochloraz-resistant strain was significantly greater than in the sensitive strain, and then the *CYP55A3* gene was knocked out from the resistant strains, and also complemented to understand its functions. Subsequent studies also show that the sensitivity to prochloraz was significantly increased in the knockout mutant (∆*Ltcyp55a3*) after the knockout of the *CYP55A3* gene, with a 9.17-fold increase. The sensitivity of the complemented mutant (∆*Ltcyp55a3*/*LtCYP55A3*) was similar to that of the wild-type, both exhibiting resistance to prochloraz. The gene expression level of the knockout mutant strain was significantly lower than that of the wild-type strain and the complemented strain, with a 16.67-fold reduction. This suggests that CYP55A3 may play a role in detoxification of prochloraz. This was similar to previous studies in the metabolic resistance which found that the pathogens upregulated the expression of P450 gene to increase resistance to fungicides [23,24,25].

According to previous reports, the synergism of detoxifying enzyme inhibitors with fungicides contributes to the study of the resistance mechanism of pathogens, and the metabolism of fungicides can be determined by monitoring the accumulation of fungicides in mycelia [27,33]. After treatment with diphenylamine (SYP-14288) for 3 h, the content of SYP-14288 in the resistant strain of *R. solani* decreased sharply, and the expression of the P450 gene increased significantly [25]. In this study, P450 gene *CYP55A3* mediating prochloraz resistance was also verified by the following test results. The results showed that the P450 enzyme activity of the complemented strain was significantly greater than that of the knock-out mutant strain. On the contrary, the accumulation of prochloraz in the complemented strain was significantly lower than that in the knock-out mutant strain after treatment with prochloraz. Additionally, the effect of the P450 inhibitors 1-ABT on the in vitro activity of prochloraz against *L. theobromae* strains was studied. The inhibition increased significantly when treated with the combination of prochloraz and 1-ATB for the complemented strain. All these results indicated that *CYP55A3* may be involved in *L. theobromae* resistance to prochloraz.

Moreover, multidrug resistance is often caused by the metabolism of fungicides, and overexpression of P450 genes can lead to the development of multidrug resistance in *R. solani*, resulting in resistance to benomyl, carbendazim and difenoconazole [34]. The study by Sang et al. (2018) revealed that the overexpression of three P450 genes (*CYP561*, *CYP65* and *CYP68*) has been identified and confirmed to contribute to resistance to multiple fungicides, which has been shown to play a role in the development of *S. homoeocarpa* resistance to fungicides of different types of action; moreover, Green et al. (2018) reported faster rates of chlorothalonil biotransformation by CYP561 and CYP65 overexpression strains [23,32]. A previous study indicated that the overexpression of the efflux transporters has been shown to be associated with the development of multidrug resistance to fungicides by *B. cinerea* [35]. In this study, the wild-type strain HL02 exhibited a multidrug resistance phenotype, and the *CYP55A3* knockout mutant was confirmed to be more susceptible to prochloraz, carbendazim and pyraclostrobin, and the accumulation of prochloraz in the knockout mutant was significantly greater than that in both of the HL02 strains. This result suggests that the overexpression of the P450 gene *CYP55A3* is associated with the development of multidrug resistance to fungicides by *L. theobromae*s.

The P450 gene *CYP55A3* was involved in the metabolism of the fungicide prochloraz, and this result was further tested via molecular docking analysis. In this study, a 3D P450 (CYP55A3) model was constructed and the optimum binding mode between CYP55A3 and prochloraz was generated on the basis of the conformations obtained via molecular docking. The homology model and molecular docking results suggest that prochloraz interacts with CYP55A3. Molecular docking analysis has been widely used to evaluate binding between proteins and chemicals and has been used to explain possible protein‒fungicide interactions [36,37]. It has been used to predict metabolism resistance to DMI in *F. graminearum*, or CYP81A68 confers metabolic resistance to herbicides in weeds [38].

The study also revealed that the fungus-specific transcription factor ShXDR1 can coordinate with cytochrome P450 and ABC transporter proteins to play a synergistic role in detoxifying xenobiotics [23]. Resistance to the uncoupler SYP-14288 in *R. solani* was shown to be associated with the overexpression of the glutathione S-transferase (GST) and P450 genes [27]. It suggests that P450, GST and ABC transporter proteins may act synergistically, leading to metabolic resistance to fungicides, but this requires further validation. Compared to insects, weeds, and bacteria, fungi possess a wider range of P450 genes, but the biological functions of most fungal P450 remain undefined. The mechanism of fungal metabolic resistance remains in the preliminary stages of study, and further exploration is needed to elucidate the specific regulatory pathways involved in the different detoxification phases.

Overall, DMI fungicide resistance involves the overexpression of target gene and/or detoxication enzyme gene. No mutation was found in the target gene *LtCYP51* of the prochloraz-resistant strain, but a higher expression of *LtCYP51* was observed in the prochloraz-resistant strain compared to the susceptible strain. And P450 detoxification metabolism plays an important role in prochloraz resistance of *L. theobromae*. This is the first report that the prochloraz resistance of *L. theobromae* was associated with the P450 gene. The overexpression of the P450 gene *CYP55A3* in *L. theobromae* affected the detoxification of fungicides, resulting in multidrug resistance. These findings further verified that resistant strains could enhance detoxification metabolism to produce resistance to fungicides. These findings suggest that metabolic resistance poses a significant threat to pesticide efficacy, as it can inherently confer resistance to existing, new, or undiscovered chemical pesticides. Our results provide new insights into the resistance mechanism of plant pathogenic fungi in response to fungicides.

## Figures and Tables

**Table 1:** Synergism of P450 enzyme inhibitors to prochloraz.

Strains	^a^ Inhibition Rates (%)		
	Pro	Pro+1-ABT	^b^ SR
HL02	56.42 b	69.45 a	1.23
∆*Ltcyp55a3*	56.02 a	49.29 b	0.88
∆*Ltcyp55a3*/*LtCYP55A3*	49.66 b	61.56 a	1.24

^a^ Values within columns followed by the same letter do not differ significantly (*p* ≤ 0.05). Pro: prochloraz, Pro+1-ABT: prochloraz and 1-aminobenzotriazole mixture. ^b^ SR: synergistic ratio = the inhibition rate of mixture/the inhibition rate of prochloraz alone.

## Data Availability

All data generated or analyzed during this study are included in this article.

## Supplementary Materials

The following supporting information can be downloaded at: https://www.mdpi.com/article/10.3390/jof10110757/s1, Figure S1: Ramachandran plot of CYP55A3 details of the final modeled structure generated by Swiss Model; Table S1: Primers utilized in this study; Table S2: Reference isolates from GenBank included in the phylogenetic analyses.
