# Supplementary material for: Exploring the Molecular Mechanisms of Resistance to Prochloraz by Lasiodiplodia theobromae Isolated from Mango
Source: J Fungi (Basel). 2024 Oct 31;10(11):757. doi: 10.3390/jof10110757 (PMC11595241; doi:10.3390/jof10110757)
Supplement: Supplementary file 1 [file jof-10-00757-s001.zip › jof-3267988-supplementary.pdf]

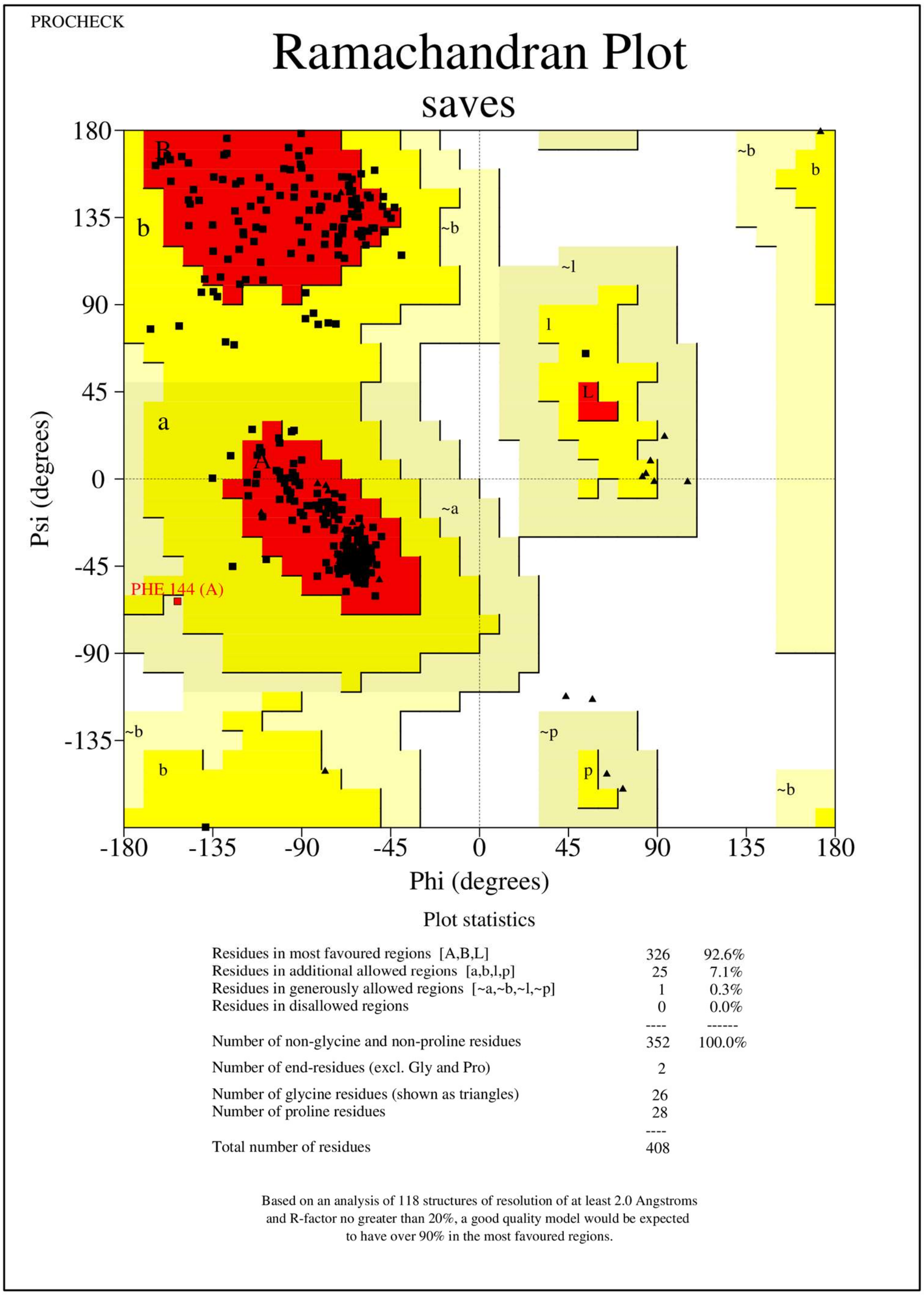

Figure S1. Ramachandran plot of CYP55A3 details of the final modeled structure generated by Swiss Model.

Table S1. Primers utilized in this study.

Primers	Sequence (5′-3′)	Description
CYP55A3-F	CAACGTCGGACCACATAAGCA	Amplifcation of the coding region of the Lt CYP55A3 gene
CYP55A3-R	ACGTGGTTGTTACCTACCCTC	
CYP55A3-CDS-F	ATGGTTTCCGACACTGTCCC	Amplifcation of the open-reading frame of the Lt CYP55A3 gene
CYP55A3-CDS-R	CTAGCCAGGGGCTTTTACGG	
UpF1	ACCATGATTACGCCAAGCTTACGAA ACTTTGCACCTTGCCT	Amplifcation of CYP55A3 upstream fragment for construction of the gene deletion vector
UpR1	CGGTGAGTTCAGGCTTTTTCATGCTG ACACGATCTTAAGTCCT	
DownF1	TCGTCCGAGGGCAAAGAAATAGCTG ATGTCGTGCAAATGGCCTT	Amplifcation of CYP55A3 downstream fragment for construction of the gene deletion vector
DownR1	CTCGGTACCCGGGGATCCGCTCCTA ATTAGGCCAAAATG	
HYG-F	AGGACTTAAGATCGTGTCAGATGAA AAAGCCTGAACTCACCG	Amplifcation of the CaMV 35s promoter and the hygromycin resistance gene fragment
HYG-R	AAGGCCATTTGCACGACATCAGCTA TTTCTTTGCCCTCGGACGA	
RT-Act Lt-F	GGAGATGAGGCACAGTCG	Amplifcation of the actin gene
RT-Act Lt-R	GCGGTGGTGGAGAAAGAGT	
RT-CYP55A3-F	CCAAACAAGCCGCCATCCG	Quantitative RT-PCR primers for analysis of CYP55A3 expression
RT-CYP55A3-R	GCCATTTGCACGACATCAGC	
RT-gliC-F	CTCGCAGCAACCTCGTACCTCT	Quantitative RT-PCR primers for analysis of gliC expression
RT-gliC-R	GCGAGACGATCCACCCGTTC	
RT-apf7-F	ATCACTGGACCGATGCCTTG	Quantitative RT-PCR primers for analysis of apf7 expression
RT-apf7-R	GCTGAAGGCGAGGTACTTGT	
RT-himC-F	AGTCGCACTTTGAGGACCTG	Quantitative RT-PCR primers for analysis of himC expression
RT-himC-R	GCTCTTGCCGAAGAGGAACT	
RT-cyb5d2-F	CTCCCGGCAGGCATTTCTAC	

RT-cyb5d2-R	GGATGTACATTTCCTCGGCG	Quantitative RT-PCR primers for analysis of cyb5d2 expression
RT-stcS-F	CCTGCCGAACAACCCATTTC	Quantitative RT-PCR primers for analysis of stcS expression
RT-stcS-R	GCCGTTGATTAGGGGCTCTT	
RT-fabG-F	AGAAGGGCTCCGTCATCAAC	Quantitative RT-PCR primers for analysis of fabG expression
RT-fabG-R	GTGGACAATGGCCAGCTCAC	
RT-ABA1-F	CAAATTCCAGGCCACGGTTG	Quantitative RT-PCR primers for analysis of ABA1 expression
RT-ABA-R	CGGCCGAGGGAACAATAACT	

Table S2. Reference isolates from GenBank included in the phylogenetic analyses.

**Species**	**Isolate**	**Host**	**Origin**	**GenBank accession number**
Macrophomina phaseolina	MPI-SDFR-AT-0080	*Arabidopsis*	USA	KAH7064874.1
Lasiodiplodia theobromae	CSS-01s	*Vitis vinifera*	China	KAF9635644.1
Lasiodiplodia theobromae	LA-SOL3	*Vitis vinifera*	Peru	KAB2574247.1
Colletotrichum siamense	CAD1	*Manihot esculenta*	Japan	KAF4862283.1
Halenospora varia	MPI-CAGE-AT-0135	*Arabidopsis*	USA	KAH6678994.1
Mytilinidion resinicola	CBS 304.34	*Larix laricina*	USA	XP_033583365.1
